# In Situ Green Synthesis of Red Wine Silver Nanoparticles on Cotton Fabrics and Investigation of Their Antibacterial Effects

**DOI:** 10.3390/ijms27020952

**Published:** 2026-01-18

**Authors:** Alexandria Erasmus, Nicole Remaliah Samantha Sibuyi, Mervin Meyer, Abram Madimabe Madiehe

**Affiliations:** 1 Nanobiotechnology Research Group, Department of Biotechnology, University of the Western Cape, Bellville 7535, South Africa; 3940022@myuwc.ac.za; 2 DSTI/Technology Innovation Agency Nanotechnology Platform, Department of Biotechnology, University of the Western Cape, Bellville 7535, South Africa; memeyer@uwc.ac.za; 3 Health Platform, Advanced Materials Division, Mintek, Randburg 2194, South Africa

**Keywords:** antibacterial activity, antimicrobial resistance, silver nanoparticles, green synthesis, ESKAPE, red wine extract

## Abstract

Antimicrobial resistance (AMR) is a major global health concern, which complicates treatment of microbial infections and wounds. Conventional therapies are no longer effective against drug resistant microbes; hence, novel antimicrobial approaches are urgently required. Silver nanoparticles (AgNPs) offer stronger antimicrobial activity, and in situ synthesis improves stability, uniformity, cost efficiency, and bioactivity while minimising contamination. These features make AgNPs well-suited for incorporation into textiles and wound dressings. Red wine extract (RW-E), rich in antioxidant and anti-inflammatory compounds was used to hydrothermally synthesise RW-AgNPs and RW-AgNPs-loaded on cotton (RWALC) by optimising pH and RW-E concentration. Characterisation was performed using UV–Vis spectroscopy, dynamic light scattering (DLS), and High Resolution and Scanning electron microscopy (HR-TEM and SEM). Antibacterial activities were evaluated against human pathogens through agar disc diffusion assay for RWALC and microdilution assay for RW-AgNPs. RWALC showed higher potency against both Gram-negative and Gram-positive bacteria, with inhibition zones of 12.33 ± 1.15 to 23.5 ± 5.15 mm, that surpassed those of ciprofloxacin (10 ± 3 to 19.17 ± 1.39 mm at 10 μg/mL). RW-AgNPs exhibited low minimum inhibitory concentrations (MIC: 0.195–3.125 μg/mL) and minimum bactericidal concentrations (MBC: 0.78–6.25 μg/mL). Preincubation with β-mercaptoethanol (β-ME) inhibited the antibacterial activity of RWALC, suggesting that thiolated molecules are involved in AgNPs-mediated effects. This study demonstrated that green-synthesised RW-AgNPs, incorporated in situ into cotton, conferred strong antibacterial properties, warranting further investigation into their mechanisms of action.

## 1. Introduction

Bacterial infections have historically caused deadly outbreaks and still remain a major health concern, particularly in the context of wound infections. These pathogens can enter the body through broken skin, leading to delayed healing, systemic illness, and increased risk of AMR [1]. Once the human body is infiltrated by bacteria, they form colonies that become pathogenic and result in serious and deadly illnesses [2,3]. Bacterial infections significantly impact global public health, and account for ~9.7% mortality [3]. Tuberculosis is the leading cause of death from bacterial illnesses, accounting for 2.3% of fatalities. Human bacterial infections impose a significant strain on the global healthcare system, leading to prolonged patient hospitalisation, steep medical expenses, and persistent illnesses [4]. The bacteria responsible for these infections can acquire drug resistance, which further presents a substantial threat to public health and development of antimicrobial agents [5]. AMR arises when microorganisms acquire the capacity to withstand the effects of antimicrobial drugs employed in the treatment of various infections [6]. AMR is caused by excessive usage and overprescription of antibiotics, and escalated by the lack of innovative antimicrobial drugs [7]. In the absence of intervention, it is widely anticipated that AMR could become one of the leading global health threats by 2050 [6]. Bacterial antibiotic resistance is a considerable challenge and has intensified with the diminished rate of development of new and effective antibiotics since the early 1990s [6]. Since the discovery of penicillin, more than 150 new antibiotics have been discovered; however, their overuse has resulted in AMR and the emergence of multidrug-resistant organisms (MDROs) [8]. In 2017, the WHO published a list of pathogens that require new antibacterial treatments and prioritised the research and development of innovative antibiotics [9]. The priority pathogens were *Enterococcus faecium*, *Staphylococcus aureus*, *Klebsiella pneumoniae*, *Acinetobacter baumannii*, *Pseudomonas aeruginosa*, and *Enterobacter* species, designated ESKAPE [9]. ESKAPE presents the greatest threat of morbidity and mortality, especially in low- and middle-income countries, resulting in a considerable global economic burden [6]. These bacteria have acquired genetic modifications that enable them to evade the effects of antimicrobial therapies [10]. At present, antimicrobial-resistant *E. coli* poses considerable clinical concern, as it adversely impacts the health of both humans and animals [9]. If AMR is not promptly and effectively managed, it may result in retrogression to the pre-antibiotics era. Even benign ailments may become lethal. Therefore, innovative strategies are urgently needed to address the issue of AMR [6]. Antibiotic therapy remains the conventional treatment for bacterial infections. Although antibiotics represent a significant advancement in medical history, their effectiveness in treating microbial infections is limited [11] by antibiotic resistance [12]. Recently discovered antibiotics, such as ceftobiprole, ceftaroline, tedizolid, and delafloxacin, have demonstrated efficacy against MDROs [13] but also have the potential to contribute to the progression of AMR. It is therefore prudent to prioritise the use of non-antibiotic therapies for the treatment of microorganisms [6].

Historically, plant-based therapies have been employed to treat bacterial infections, sometimes alongside antibiotics and other conventional treatments [11]. This is because plant extracts contain several phytochemicals with antimicrobial properties, which can suppress the proliferation of pathogens or serve as adjuvants to modulate pathogenicity [14]. As such, the development of novel antimicrobials derived from polyphenols [14,15,16], which include phenolic acids, quinones, saponins, flavonoids, tannins, coumarins, terpenoids, and alkaloids [15,17], presents a significant research opportunity. Phenolics may increase the effectiveness of antibiotics, potentially reducing the treatment dosage and eliminating pathogenic and antibiotic-resistant microbes [18]. The antibacterial activity exhibited by polyphenols is attributed to their structural composition, which may lead to differences in their antimicrobial efficacy. Polyphenol-rich extracts, such as those of grapes and their byproducts, were reported to possess antibacterial activity [19,20,21]. Compared with white grapes, red grapes and their byproducts presented higher MICs [22], as well as synergistic effects when used in combination with antibiotics [23,24]. Grapes contain organic compounds, including 3,5-dihydroxybenzoic acid, protocatechuic acid, and 4-hydroxy-5-(phenyl)-valeric acid, which are involved in plant defence against detrimental diseases [25]. Resveratrol is one of the phytochemical found in grapes that has been extensively researched due to its numerous biological actions [26]. It possesses many benefits owing to its cardioprotective, immunomodulatory, anti-inflammatory, chemopreventive [27], antibacterial, antioxidant, and anti-neurodegenerative properties [26]. Additionally, red grapes and red wine can mitigate various human diseases [28]; however, the byproducts of these valuable materials are discarded as waste following the wine production process [25,29]. Grape pomace (GP), comprising seeds, skins, stems, and leaves, is an economical and valuable source of bioactive phytochemicals that are also present in wine byproducts [25]. These compounds also have many health benefits [29] and have the potential to act as reducing and stabilising agents in the green synthesis of metallic NPs (MNPs).

Nanotechnology has advanced many industries in recent years, worldwide. It has established itself as a promising technology of the contemporary century, with a significant emphasis on integrating nanotechnology methods and products into various applications including the medical field [30]. MNPs exhibit unique and distinctive biological characteristics, making them essential in pharmaceutical applications. AgNPs are important and intriguing MNPs that are useful in numerous applications [31]. Substantial evidence indicates that Ag^+^ and Ag-based compounds have a potential to eradicate bacteria, suggesting that they could serve as alternative treatment for AMR [32]. Green nanotechnology integrates green concepts in the development of eco-friendly procedures that mitigate potential risks to the environment and human health [33]. Several studies have produced biogenic AgNPs utilising microorganisms and plant extracts [34,35,36]; synthesis using plant extracts is a straightforward, economical, and eco-friendly approach [37]. Green-synthesised AgNPs have demonstrated broad-spectrum antimicrobial capabilities with the potential to treat or prevent bacterial and fungal infections [38] in wounds and diseases [39]. Biogenic AgNPs could serve as alternative antimicrobial agents to address AMR [40], as MNPs use different mechanisms than conventional antimicrobial agents and are generally considered less prone to inducing AMR [41]. While some studies suggest that under certain conditions, sub-lethal NP exposure may contribute to adaptive bacterial responses, AgNPs remain promising due to their multi-target antimicrobial mechanisms, which reduce the likelihood of resistance development [41,42]. AgNPs have been extensively utilised in clinical environments and medical research, with AgNPs-based products now available in commercial space [42]. AgNPs can be integrated with other systems to create novel nanomaterials and have emerged as an attractive strategy for creating alternative antimicrobial agents [43]. These hybrid materials exhibit the antibacterial properties of AgNPs by releasing Ag^+^ that infiltrates microbial cells and disrupts DNA replication [43]. Thus, employing AgNPs to enhance textile (cellulosic) materials has emerged as an attractive strategy for creating alternative therapies for microbial infections [43].

Cotton has served as a crucial material in medical applications for many years, with its use recorded as early as the Middle Ages, primarily for wound dressing [44]. The intrinsic characteristics of cotton, such as its softness, breathability, and high absorption, make it an essential material in wound care and management [45]. With advancements in medical knowledge, especially owing to the introduction of antiseptic techniques and the germ theory of disease, the use of cotton in medical applications has markedly increased [43]. The adaptability of cotton has facilitated its integration into various medical goods, such as sterile gauze, bandages, and surgical dressings. These applications leverage the capacity of cotton to act as a barrier against microbial contamination while efficiently controlling wound exudates [46]. The hypoallergenic properties of cotton highlight its appropriateness in medical settings, especially for individuals with delicate skin or those susceptible to allergic reactions [47]. This attribute, along with its biodegradability and natural origin, renders cotton a superior option compared with synthetic materials, particularly in light of growing environmental and sustainability issues in healthcare [48]. Recent breakthroughs in cotton processing and treatment have resulted in the creation of new medical textiles that preserve the traditional advantages of cotton while integrating contemporary technical improvements [49]. The use of plant extracts in the green synthesis of AgNPs is considered a safe, cost-effective, and environmentally sustainable approach for producing antibacterial coatings and textiles, with AgNPs-functionalized cotton fabrics demonstrating significant potential in the biomedical sector [50].This study aimed to synthesise RW-AgNPs and RWALC via a hydrothermal method and investigate their antibacterial properties.

## 2. Results and Discussion

### 2.1. Hydrothermal Synthesis of RW-AgNPs and RWALC

**Figure 1:**
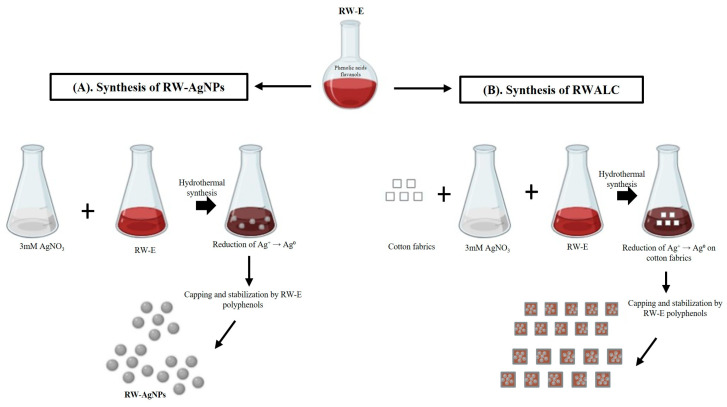
Schematic illustration of hydrothermal green synthesis of RW–mediated AgNPs via phytochemical reduction in Ag^+^ ions (**A**), and in situ NP formation on cotton fabrics (RWALC) (**B**).

Hydrothermal synthesis of RW-AgNPs and RWALC is depicted in Figure 1, aqueous RW-E was used as reducing and capping agents, with AgNO_3_ serving as a precursor. The polyphenolic compounds present in RW-E reduced and stabilised RW-AgNPs in solution (Figure 1A), as well as in textiles (Figure 1B). RW contains polyphenols such as resveratrol, catechins, anthocyanins, and flavonoids, which were involved in the synthesis of MNPs. While detailed phytochemical profiling of RW-E was not performed in this study, these compounds are well documented in the literature [51].

**Figure 2:**
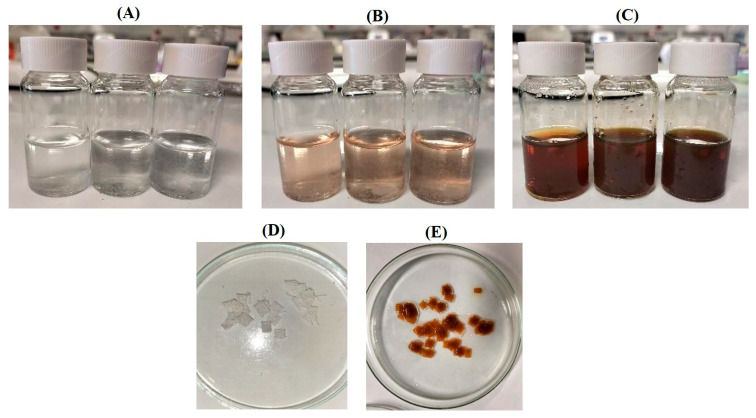
Hydrothermal synthesis of RW-AgNPs and RWALC. (**A**) AgNO_3_ solution, (**B**) mixture of AgNO_3_ and RW-E, (**C**) reaction mixture at the end of RW-AgNPs synthesis, (**D**) cotton fabrics before and (**E**) after in situ synthesis of RW-AgNPs on cotton fabrics.

During the synthesis process, the mixture of RW-E with AgNO_3_ changed to a brown solution, indicating the formation of RW-AgNPs in the solution (Figure 2A–C). The in situ synthesis of RWALC was indicated by the colour change in the cotton fabric from white to yellowish-brown, as shown in Figure 2D,E. The change in colour of the cotton fabrics from white to yellowish-brown suggested the presence of AgNPs on the surface of the cotton fabrics. The colour change is attributed to the surface plasmon resonance (SPR) and the bioreduction of Ag^+^ by phytochemicals present in the RW-E [52].

The hydrothermal synthesis of RWALC was evaluated at a natural pH of 4.3 and at pH 10 using 3 mM AgNO_3_, which was previously optimised during the synthesis of RW-AgNPs [53]. The resulting RWALC was visually assessed through colour changes; at pH 4.3 RWALC had a strong yellowish hue, while the cotton fabrics at pH 10 displayed a dark brown hue (Figure S1). The differences in the colour of the cotton fabrics suggested that the RW-AgNPs formed at different pH values had different properties, especially in terms of concentration and size. Acidic pH values typically produce lower yields of NPs compared to alkaline pH. This is attributed to the limited ionisation of functional groups in RW-E phytochemicals at acidic pH, which hinders the reduction in Ag^+^ due to minimal interaction with the cotton fabrics [54]. At alkaline pH, the ionisation of phytochemicals is enhanced, increasing their ability to interact with the metal precursor. Alkalinity facilitates conversion of Ag^+^ ions to Ag^0^ and promotes binding of AgNPs to the cotton fibres. Deprotonation of RW-E functional groups at pH 10 contributes to improved stability and dispersity of RW-AgNPs, which likely aids in achieving more uniform NPs deposited onto the cotton fabrics [54,55]. As a result, the cotton fabrics subjected to alkaline pH not only appeared darker in colour but also loaded higher amount of smaller, well-distributed RW-AgNPs than the cotton fabrics at acidic pH, as shown in Figure S1 [54,56].

**Figure 3:**
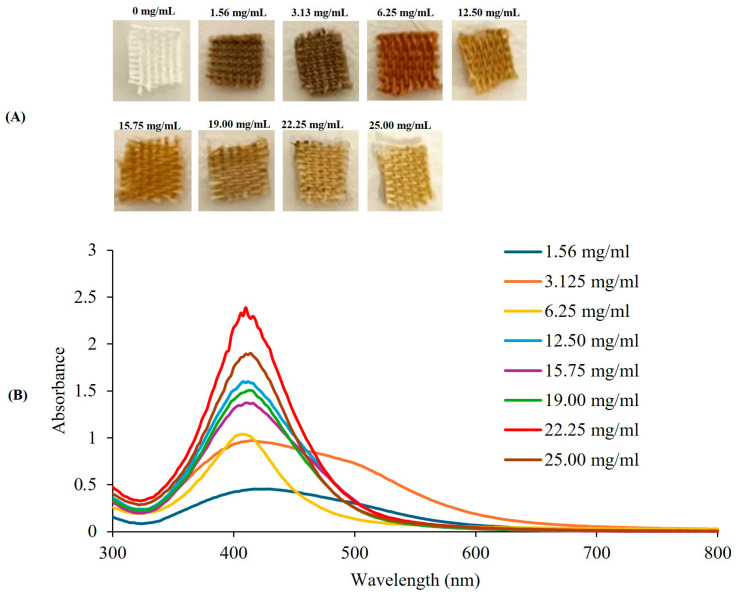
Effects of the RW-E concentration on the hydrothermal synthesis of RWALC using 3 mM AgNO_3_ at pH 10. (**A**) RWALC synthesised with various concentrations of RW-E and (**B**) UV–Vis spectra of RW-AgNPs.

Hydrothermal synthesis of RWALC at increasing RW-E concentrations significantly altered the properties of the AgNPs, as evidenced by the different colours of the cotton fabrics (Figure 3A). The UV–Vis spectra confirmed the conversion of Ag^+^ to Ag^0^ by RW-E, as well as the synthesis of RW-AgNPs at all RW-E concentrations (Figure 3B). At lower concentrations (1.56, 3.125, and 6.25 mg/mL), a darker brown colour was observed on the cotton fabrics, whereas a lighter yellow colour was observed at higher concentrations. RW-AgNPs synthesised at 1.56 and 3.25 mg/mL presented the lowest yields, which is attributed to the inadequate availability of phytochemicals required to completely reduce Ag^+^ to Ag^0^ [57], resulting in the formation of larger NPs. RW-E at 1.56 and 3.25 mg/mL produced very broad spectra, suggesting the presence of anisotropic and polydispersed RW-AgNPs. Distinct AgNPs spectra were observed at 6.25 to 25 mg/mL, and the highest yield was at 22.25 mg/mL. The absorption peak of the RW-AgNPs synthesised at 22.25 mg/mL was at 410 nm, with an absorbance value of 2.388, whereas those synthesised at 19 and 25 mg/mL presented λ_max_ values at 414 nm, with corresponding absorbance values of 1.506 and 1.902, respectively. In contrast, the resulting RWALC exhibited a light-yellow colour, suggesting minimal deposition of RW-AgNPs, while the majority remained in solution. The concentration of RW-E that produced the darkest hue of the RWALC was 6.25 mg/mL. Analysis of the SPR band at 6.25 mg/mL revealed a λ_max_ of 408 nm and an absorbance of 1.036. Although 6.25 mg/mL presented one of the lowest absorbances in this experiment, the λ_max_ of RW-AgNPs at 6.25 mg/mL demonstrated the most pronounced blue shift, indicating the formation of the smallest RW-AgNPs among all RW-E concentrations.

Following thorough evaluation, 6.25 mg/mL RW-E was determined to be the optimal concentration for RWALC synthesis, as it yielded RW-AgNPs with the most uniform sizes and resulted in the most significant colour change in the cotton fabrics (Figure 3A). These findings differ from those of Rajput et al. (2025) [58], who reported a gradual darkening of cotton fabrics following treatment with increasing concentrations (5–15%) of pomegranate rind extract. A similar trend was observed with increasing quantities of AgNPs synthesised from eucalyptus extract [59]. Higher extract concentrations resulted in increased colour strength (K/S values), indicating more profound colouration. The extract improved the binding and homogeneous distribution of the AgNPs [58]. In this investigation, higher RW-E concentrations resulted in lighter-coloured fabrics. This disparity could be attributed to differences in the preparation of the NP-loaded fabrics—specifically, in situ reduction in NPs directly on the cotton fabrics by the plant extracts versus dyeing the fabrics with pre-synthesised biogenic AgNPs, type of extract, as well as variations in NP size, charge, and surface chemistry; all of which influence interactions of NPs with the fabrics [58,60].

### 2.2. Upscaled Synthesis and Characterisation of RW-AgNPs and RWALC

**Figure 4:**
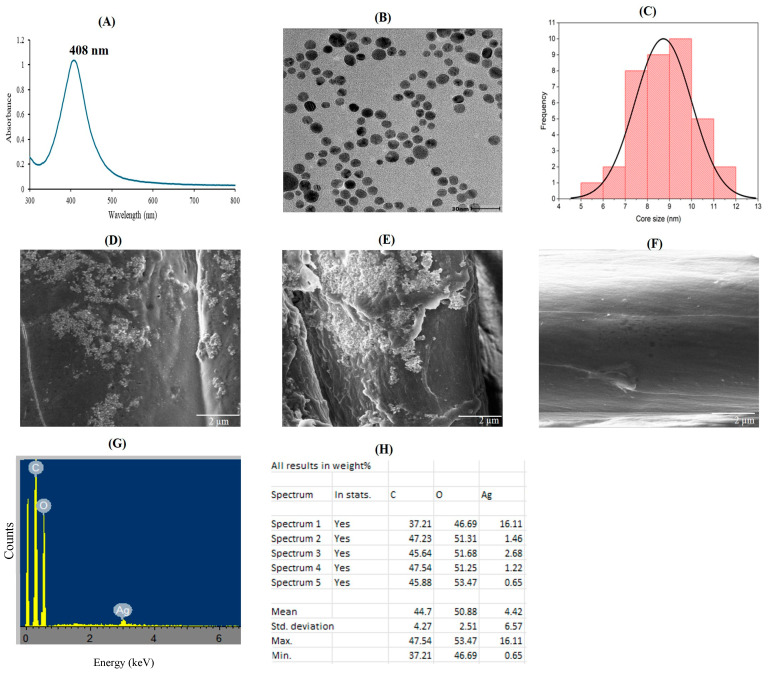
Physicochemical properties of the RW-AgNPs and RWALC. (**A**) UV–Vis, (**B**) HR-TEM, and (**C**) core size distributions of RW-AgNPs. (**D**,**E**) SEM micrographs of the RWALC and (**F**) untreated cotton fabric. (**G**) EDX spectrum and (**H**) elemental composition of the RWALC.

A large-scale synthesis of RW-AgNPs and RWALC was conducted using previously optimised conditions, and the resulting products were characterised via several physicochemical methods. The UV–Vis absorption spectra of the upscaled and optimised RW-AgNPs is displayed in Figure 4A. A spectrum with an SPR peak at 408 nm confirmed the synthesis of uniform sized and spherical RW-AgNPs. The absorbance value of 1.24 demonstrated a significant amount of RW-AgNPs in solution (Table 1). The DLS data presented in Table 1 revealed that the RW-AgNPs had a hydrodynamic size of 104.30 ± 5.51 nm, a polydispersity index (PDI) value of 0.344 ± 0.2, and a ζ-potential of −11 ± 1.5 mV. HR-TEM confirmed that the RW-AgNPs were spherical and mostly monodispersed (Figure 4B), which was consistent with the UV–Vis spectrum and the PDI value. The core size range of the RW-AgNPs was 5–11 nm, with an average core size of 9.2 ± 1.5 nm (Figure 4C). The in situ-synthesised RWALC was analysed via SEM and energy-dispersive X-ray (EDX) spectroscopy to evaluate the deposition of RW-AgNPs on the cotton fabric, as well as the elemental compositions of both the unloaded cotton fabric and the RWALC (Figure 4D–F). The SEM micrographs of RWALC demonstrated the presence of spherical RW-AgNPs at a reduced quantity distributed across the cotton fabric. The EDX spectrum showed a minor peak for Ag (Figure 4G) at an optical absorption peak near 3 keV, indicating AgNPs characteristic SPR, as determined by the AgLα lines [61]. The EDX component analysis in Figure 4H indicated that the sample was predominantly composed of carbon (44.7%) and oxygen (50.88%), which are essential components of cellulose in the cotton fabric [43]. Cellulose constitutes more than 90% of cotton textiles. The sample also contained 4.42% Ag, which was attributed to the adherence of RW-AgNPs to the cotton. In contrast, the control cotton fabric contained no Ag.

### 2.3. TPC Analysis of RW-E and RW-AgNPs

RW-AgNPs surprisingly had higher TPC than RW-E. As shown in Table 2, the TPC of RW-AgNPs was 121.17 ± 7.93 μg GAE/mL, whereas the TPC of RW-E was 31.273 GAE/mL, which was a fourfold increase. This finding indicated that the phenolic compounds found in RW-E participated in reducing, stabilising, and capping the RW-AgNPs during the synthesis process. The TPC derived from *Vitis vinifera* red GP was reported to be 54.26 ± 1.66 μg GAE/g [19], whereas the white GPs from Cabernet Franc and Chambourcin were 153.8 ± 1.83 and 92.0 ± 2.16 μg GAE/g, respectively [19].

### 2.4. Antioxidant Activities of RW-E and RW-AgNPs

**Figure 5:**
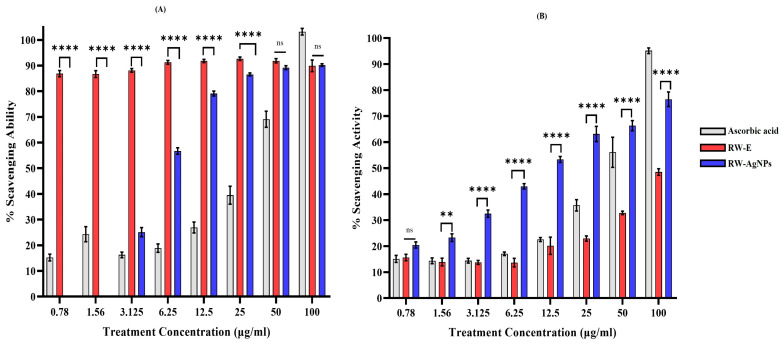
Antioxidant activity of RW-E and RW-AgNPs. (**A**) DPPH and (**B**) ABTS radical scavenging ability of RW-E, RW-AgNPs, and ascorbic acid. The data are presented as the means ± SEMs (*n* = 9). Statistical significance was determined via two-way ANOVA, where ns = not significant, ** = *p* < 0.01, and **** = *p* < 0.0001.

The antioxidant properties of RW-E and RW-AgNPs were assessed via DPPH and ABTS assays. The DPPH scavenging properties of the test samples were compared with those of ascorbic acid. As illustrated in Figure 5A, RW-E exhibited scavenging activity exceeding 85% across all the tested concentrations (0.78–100 μg/mL), with activity levels exceeding those of ascorbic acid. The DPPH scavenging activity of RW-AgNPs increased in a dose-dependent manner, with no activity detected at lower concentrations of 0.78 and 1.56 μg/mL. RW-AgNPs had an IC_50_ value of 4.67 μg/mL (Figure 5A). While the RW-AgNPs demonstrated significant activity, their efficacy was lower than that of RW-E and ascorbic acid. These findings indicated that the AgNPs derived from RW-E demonstrated hydrogen-donating properties and acted as antioxidants. The antioxidant activity of RW-E and RW-AgNPs could be attributed to the phytochemicals present in RW-E [59]. At a concentration of 100 μg/mL RW-AgNPs, the DPPH free radical scavenging activity was approximately 79.16%, which was comparable to the 84.60% of ascorbic acid [62]. The reduced DPPH radical scavenging activity observed for RW-AgNPs compared to RW-E could be due to the consumption of RW polyphenols during the formation of NPs [29]. These phytochemicals, which are primarily responsible for hydrogen atom donation in DPPH assays, are most likely involved in the reduction and capping of Ag^+^, thus reducing their availability for free radical scavenging [63].

RW-E, RW-AgNPs and ascorbic acid also showed dose-dependent ABTS^•+^ radical scavenging activity (Figure 5B). However, among the three samples, RW-E demonstrated the lowest efficacy. RW-AgNPs exhibited greater scavenging activity than ascorbic acid did at the specified concentrations (0.78–50 μg/mL). At 100 μg/mL, ascorbic acid resulted in the highest antioxidant activity, with a scavenging activity of 95.2%, which was greater than that of RW-E (48.5%) and RW-AgNPs (76.5%). The IC_50_ values for RW-AgNPs, RW-E, and ascorbic acid were 8.29 μg/mL, 119.27 μg/mL, and 46.79 μg/mL, respectively. Previous studies indicated that majority of plant extracts synthesised AgNPs had greater antioxidant activity compared to their corresponding plant extracts, due to the synergistic effects between RW-E phytochemicals and the NP metal core, which aligns with the outcomes of the present study [64]. Khuda et al. reported that the % ABTS scavenging activity observed at a concentration of 1000 μg/mL was 61% for the crude extract, 74% for the AgNPs, and 80% for the positive control. The IC_50_ values for each sample were 25.45, 18.88, and 15.34 μg/mL, respectively [65]. In contrast to the results of the DPPH assay, RW-AgNPs displayed high radical scavenging activity for ABTS, which can be explained by the different reaction mechanisms of the two assays. Unlike DPPH, ABTS scavenging involves transfer mechanisms for both electrons and hydrogen atoms, which could be enhanced by the presence of AgNPs and surface-bound phenolic compounds [19].

### 2.5. Antibacterial Activity of RW-AgNPs

The antibacterial activity of the RW-AgNPs was evaluated on Gram-negative and Gram-positive strains via a microdilution assay to assess the MIC and MBC of RW-AgNPs. RW-AgNPs exhibited broad-spectrum antibacterial activity against the selected bacterial strains, as demonstrated in Table 3. The inhibitory effects on bacterial growth were dose dependent. *S. aureus*, followed by *E. coli* and *K. pneumoniae*, were the most susceptible to the effects of RW-AgNPs, as demonstrated by an MIC ranging from 0.195 to 1.56 μg/mL and an MBC ranging from 0.78 to 3.125 μg/mL, indicating that remarkably low concentrations of RW-AgNPs are necessary to inhibit bacterial growth and eliminate 99.9% of the bacteria. This is significant, as *S. aureus* is resistant to multiple antibiotics and is associated with various hospital-acquired infections and elevated mortality rates [66]. The lower MIC observed against *S. aureus* compared to *E. coli* may be attributed to differences in cell wall architecture. While *E. coli* possesses a thinner peptidoglycan layer, its outer membrane containing lipopolysaccharides can act as an effective cellular barrier to NPs uptake and entry [67]. In contrast, the absence of an outer membrane in Gram-positive *S. aureus* allows for more direct interaction between AgNPs and the bacterial cell wall and membrane, enhancing antibacterial efficacy. RW-AgNPs demonstrated marginally reduced efficacy against MRSA, with MIC and MBC values of 1.56 μg/mL and 3.125 μg/mL, respectively. This outcome was expected because MRSA is a multidrug-resistant strain of *S. aureus* that is resistant to all β-lactam antibiotics [68]. RW-AgNPs also demonstrated the lowest efficacy against *E. cloacae* and *P. aeruginosa*, with an MIC of 3.125 μg/mL, and the highest MBC of 6.25 μg/mL was observed for *P. aeruginosa*. The ability of RW-AgNPs to kill global high priority pathogens [1] without discriminating against Gram-negative and Gram-positive strains at concentrations closely related to those of antibiotic-based therapy. This offers a new paradigm for novel antimicrobial agents [69]. The strains under study, particularly *P. aeruginosa* and *E. cloacae*, are known to cause infections in immunocompromised patients who are at increased risk of acquiring infections mediated by these pathogens [9]. The strains under study produce extended-spectrum β-lactamases, which make them confer resistance to nearly all antibiotics, with the exception of colistin [70]. Colistin is considered the last-resort antibiotic and has high efficacy against various bacterial strains [70]. The bactericidal effect of RW-AgNPs on the test strains was notably at a very low concentration (<6.3 μg/mL).

This study revealed that RW-AgNPs had MIC values below 1 mg/mL, highlighting their remarkable antibacterial potency. Thus, these NPs present a viable alternative for treating bacterial infections that require urgent intervention [71]. The findings of the present study are corroborated by those of previous studies; for example, biogenic *Aloe vera*-AgNPs inhibited the growth of bacterial strains (*S. aureus*, *E. coli*, *A. baumannii*, and *P. aeruginosa*) and fungi [72], although at concentrations that were more than 10 times greater than those of RW-AgNPs. The antibacterial activity of RW-AgNPs was similar to that of a broad-spectrum antibiotic (ciprofloxacin), which was administered at 10 μg/mL for all bacteria, with the exception of *E. coli*, which was treated with 5 μg/mL. The MIC and MBC values of RW-AgNPs were notably lower than the concentrations of ciprofloxacin used in the microdilution method. These findings indicated that RW-AgNPs are adequate and have the potential to generate antibacterial effects comparable to those of ciprofloxacin against all the tested pathogens. These findings demonstrated the broad-spectrum antibacterial activity of RW-AgNPs, indicating that they may be more effective than traditional antibiotic therapy.

### 2.6. Evaluating the Antibacterial Activity of RWALC and Abrogation of the RWALC by β-ME

**Figure 6:**
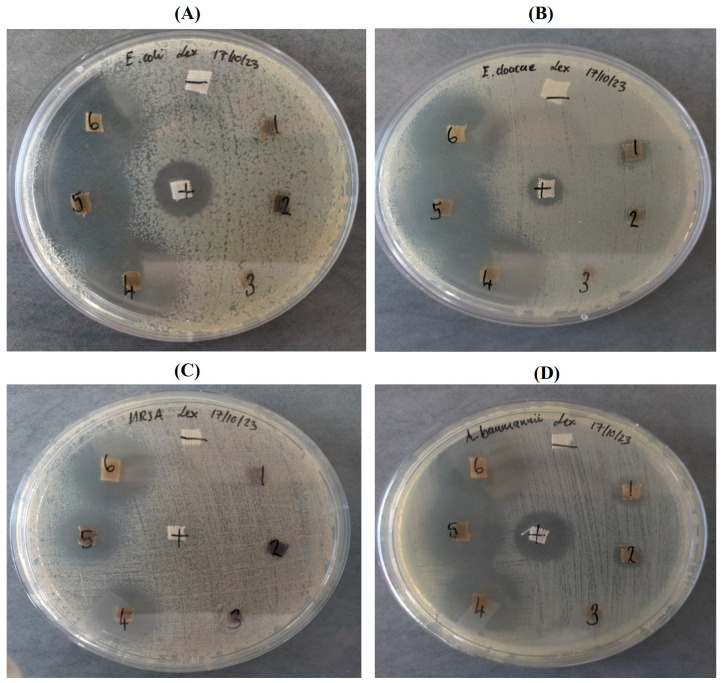
Antibacterial activity of the RWALC on selected bacteria via an agar disc diffusion assay. The bacteria were exposed to either RWALC preincubated with β-ME (1–3) or RWALC (4–6). (**A**) *E. coli*, (**B**) *E. cloacae*, (**C**) MRSA, and (**D**) *A. baumannii*. − = negative control, + = ciprofloxacin.

RWALC also demonstrated enhanced antibacterial efficacy against the test strains as shown in Figure 6, this was an important finding as they are clinically relevant strains. The RWALC treatments demonstrated higher efficacy against Gram-negative bacteria than Gram-positive bacteria. *E. cloacae* and *A. baumannii* presented the greatest susceptibility, with ZOIs of 22.17 ± 1.89 mm and 23.56 ± 2.57 mm, respectively (Figure 6B,C). Notable ZOIs were recorded against *E. coli* (Figure 5A) at 19.17 ± 3.33 mm, *K. pneumoniae* (Figure S2B) at 17 ± 2.65 mm, and *P. aeruginosa* (Figure S2C) at 14.67 ± 2.08 mm. Among the Gram-positive bacteria, MRSA (Figure 6C) and *S. aureus* (Figure S2A) exhibited ZOIs of 13.75 ± 4.60 mm and 12.33 ± 1.15 mm, respectively. These results indicated that the RWALC possesses broad-spectrum antibacterial activity, with varying degrees of efficacy depending on the bacterial strain. While some Gram-negative strains showed greater susceptibility, measurable inhibition was also achieved against AMR Gram-positive species. RWALC treatments also resulted in greater antibacterial activity than ciprofloxacin at 10 μg/mL. In a similar study by Tooklang et al. (2024) [60], AgNPs-loaded cotton fabrics via hydrothermal method presented dose-dependent activity, ZOIs increased with increasing AgNPs concentrations [60]. The ZOIs ranged from 17.83 to 21.50 mm against *E. coli* and 17.50–21.33 mm against *S. aureus* at AgNPs concentrations ranging from 250 to 2000 µg/mL. Notably, at the highest concentration (2000 µg/mL), the ZOI values approached those of the positive control (streptomycin), underscoring the potent bactericidal properties of AgNPs-coated textiles [60] and further corroborating the results observed in this study.

To demonstrate that the antibacterial properties of RW-AgNPs stem from the capacity of Ag to bind to sulfhydryl-containing molecules, RWALCs were treated with β-ME and subsequently exposed to the test strains via a disc diffusion assay. As shown in Figure 6, the RWALC preincubated with β-ME demonstrated no antibacterial activity against any of the strains as no ZOIs were detected in the agar plates compared to the untreated RWALCs (Table 4). This indicated that thiol-containing molecules such as β-ME can inhibit the antibacterial efficacy of RW-AgNPs. A study by Lethongkam et al. (2023) [73] demonstrated that β-ME and cysteine, at concentrations exceeding 17.46 and 31.25 μg/mL, respectively, effectively negated the antibacterial properties of Ag-containing compounds against *S. aureus* and *P. aeruginosa* [73]. β-ME inhibits the antibacterial properties of AgNPs via multiple mechanisms, such as surface interactions, the inhibition of ion release, the scavenging of reactive oxygen species (ROS), and alterations of their surface charge [73]. The thiol group in β-ME has a strong affinity for Ag, facilitating its interaction with the reactive AgNPs. Binding of β-ME to RW-AgNPs inactivates the NPs and obstructs their active sites, which are crucial for induction of ROS and the release of Ag^+^ ions. Both factors are crucial for the antibacterial efficacy of AgNPs [74]. Furthermore, β-ME can react with Ag^+^ ions, leading to the formation of Ag-S complexes. This reaction diminishes the availability of Ag^+^ and mitigates its toxicity to bacterial cells [73]. β-ME decreases the dissolution of AgNPs by stabilising them, thereby restricting the release of Ag^+^ that is responsible for their antibacterial activity. β-ME functions as an antioxidant and scavenges ROS, thus mitigating the oxidative stress induced by AgNPs in bacterial cells [75]. The interaction between β-ME and AgNPs may influence the ζ-potential of the NPs, in turn affecting their surface charge [76]. This modification can inhibit the interaction between AgNPs and bacterial cell membranes, thereby reducing the ability of the NPs to attach to and penetrate bacterial cells. The antibacterial effectiveness of RW-AgNPs could be significantly reduced by the presence of β-ME via any of the abovementioned mechanisms [73].

## 3. Materials and Methods

### 3.1. Methods

#### 3.1.1. Preparation of RW-E

Nederburg Baronne, a South African RW, was purchased from a local store, and the extract was prepared as previously described [77]. Briefly, the RW was subjected to rotary evaporation via a BUCHI rotary evaporator (Flawil, Switzerland), and the resulting paste was oven-dried overnight at 50 °C in an IncoTherm oven (Labotec, Cape Town, South Africa). The dried extract was transferred to 50 mL Greiner tubes and freeze-dried via a VirTis freeze dryer-BenchTop Pro with Ominitronics (SP Scientific, Warminster, PA, USA). Stock solutions of 100 mg/mL RW-E were prepared in deionised water and stored at 4 °C until use.

#### 3.1.2. Hydrothermal Synthesis of RW-AgNPs and RWALC

RW-AgNPs were synthesised via a green, phytochemical-mediated reduction process using RW-E as the reducing and stabilising agent. Briefly, an aqueous solution of 6.25 mg/mL RW-E at pH 4.3 and 10 was combined with 3 mM AgNO_3_ (Merck, Johannesburg, South Africa), at a 1:9 (*v*/*v*) ratio of RW-E to AgNO_3_. The reaction mixture was autoclaved for 20 min under standard conditions (121 °C, 15 psi) to facilitate hydrothermal synthesis. The formation of RW-AgNPs was visually indicated by a characteristic colour change and confirmed by ultraviolet–visible (UV–Vis) spectroscopy.

The synthesis of RW-AgNP-loaded cotton fabrics (RWALC) was performed following conditions previously described for AgNPs-loaded textiles, with modifications [48]. The cotton fabrics, cut into 5 mm × 5 mm fragments, were added in the mixture containing 6.25 mg/mL RW-E and 3 mM AgNO_3_ at a 1:9 (*v*/*v*) ratio. A control reaction mixture was prepared without AgNO_3_. The mixtures were autoclaved for 20 min under standard conditions (121 °C, 15 psi) for in situ hydrothermal synthesis of RWALC. Only the RW-E concentration was optimised at 1.56–25 mg/mL at natural pH and pH 10. The effective synthesis of RWALC was validated by the colour change in the cotton fabrics and liquor to a light yellow or brown solution.

#### 3.1.3. Characterisation of RW-AgNPs and RWALC

The RW-AgNPs and RWALC were characterised by UV–Vis spectroscopy via a POLARstar Omega plate reader (BMG Labtech, Ortenberg, Germany), DLS via a Zetasizer–Nano-ZS90 system (Malvern Instruments, Worcestershire, UK), high-resolution transmission electron microscopy (HR-TEM) using FEI Tecnai G2 20 FEG (FEI, Hillsboro, OR, USA), and scanning electron microscopy (SEM; FEI, Hillsboro, OR, USA) at the Electron Microscope Unit (University of Cape Town, South Africa). The core size of the AgNPs was calculated from the HR-TEM images via ImageJ software 1.53v (National Institutes of Health, Bethesda, MD, USA).

#### 3.1.4. Analysis of the Phytochemical Composition of RW-E and RW-AgNPs

The total phenolic content (TPC) of RW-E and RW-AgNPs were quantified by the Folin–Ciocalteu test, with gallic acid used as a standard. The experiment involved the addition of 20 μL of each sample (RW-E or RW-AgNPs), and gallic acid (Sigma-Aldrich, St. Louis, MO, USA) to a 96-well plate, followed by the addition of 100 μL of 10% Folin–Ciocalteu solution and incubation at room temperature for 5 min. Subsequently, 80 μL of 7.5% Na_2_CO_3_ (Sigma-Aldrich) solution was added to the wells, and the plate was incubated at room temperature for 30 min. The absorbance was read at 765 nm on a plate reader. The TPC was measured as μg gallic acid equivalent/mL on the basis of the gallic acid standard curve. The experiment was performed in triplicate and repeated three times.

#### 3.1.5. Antioxidant Activity of RW-E and RW-AgNPs

The antioxidant activities of RW-E and RW-AgNPs were assessed via two of the most common colorimetric assays, namely, 2,2-Diphenyl-1-picrylhydrazyl (DPPH) and 2,2′-azinobis-(3-ethylbenzothiazoline-6-sulfonic acid) (ABTS).

##### DPPH Scavenging Assay

The total antioxidant potential of the RW-E and RW-AgNPs was determined via the DPPH scavenging assay following a method described elsewhere [69,78]. Briefly, 100 μL of DPPH solution (0.25 mM in methanol; Sigma-Aldrich) was added to a 96-well plate and mixed with 20 μL of 0–100 μg/mL RW-E, RW-AgNPs, or ascorbic acid. The plate was incubated in the dark at room temperature for 1 h. The DPPH radical scavenging activity was determined by Equation (1):

Radical scavenging (%) = (*OD*
*blank* − *OD*
*sample*)/*OD*
*blank* × 100% (1)

##### ABTS Scavenging Assay

The antioxidant potential of RW-E and RW-AgNPs was also assessed via the ABTS assay, as described previously [69]. Briefly, 7 mM ABTS (ThermoFisher Scientific, Kandel, Germany) was mixed with 2.45 mM K_2_S_2_O_8_ (Sigma-Aldrich) at a 1:1 ratio and incubated at room temperature in a dark overnight. The blue-green ABTS solution was diluted with ethanol to obtain an absorbance of 0.70 at 734 nm. Different concentrations of RW-E, RW-AgNPs, and ascorbic acid were prepared, and 20 μL of each sample was mixed with 180 μL of ABTS in a 96-well plate. The samples were incubated at room temperature for 5 min. Thereafter, the absorbance was read at 517 nm, and the ABTS radical scavenging activity was determined via Equation (1).

#### 3.1.6. Antibacterial Efficacy of RW-AgNPs and RWALC

The antibacterial activities of RW-AgNPs and RWALC were assessed against the Gram-negative bacteria: *Escherichia coli* (*E. coli*; ATCC 35218), *Enterobacter cloacae* (*E. cloacae*; ATCC 13047), *Acinetobacter baumannii* (*A. baumannii*; ATCC 19606), *Klebsiella pneumoniae* (*K. pneumoniae*; ATCC 13883) and *Pseudomonas aeruginosa* (*P. aeruginosa*; ATCC 27853) and the Gram-positive bacteria: *Staphylococcus aureus* (*S. aureus*; ATCC 25923) and methicillin-resistant *Staphylococcus aureus* (MRSA; ATCC 33591) via agar disc diffusion and microdilution assays as described previously [79,80]. All the bacterial strains were purchased from (American Type Culture Collection, Manassas, VA, USA) and were grown to a turbidity standard of 0.5 McFarland (equivalent to 1.5 × 10^8^ CFU/mL) and used at 1:150 dilutions prepared in fresh Müeller–Hinton Broth.

##### Determination of the MIC and MBC

The MIC and MBC of the RW-AgNPs were determined by the microdilution assay as described previously [79]. Microbial cultures were prepared in accordance with the 0.5 MacFarland turbidity standard, equivalent to approximately 1.5 × 10^8^ CFU/mL, and used in a 1:150 dilution using fresh Müeller–Hinton Broth. Then, 50 μL of the bacterial suspensions were added to 96-well flat-bottom microtiter plates. For the treatments, 50 μL of RW-AgNPs at 0.195–25 μg/mL were added to the wells. For the negative control, 50 μL of Müeller–Hinton broth was added to each well. Ciprofloxacin was used as the positive control at 10 μg/mL for all strains, 5 μg/mL ciprofloxacin was used for *E. coli*, due to its higher susceptibility to the drug. The plates were incubated at 37 °C for 24 h. After incubation, the plates were visually inspected, and the MIC was identified as the minimum concentration at which no microbial growth was detected in the wells.

The MBC was determined by subculturing the bacteria-RW-AgNPs suspension onto Müeller–Hinton agar plates, starting with the wells that exhibited no obvious bacterial growth or the MIC. The plates were incubated at 37 °C for 24 h. The MBC was identified as the minimum concentration at which no growth was observed on the MHA plates. The experiments were performed in triplicate and conducted three times.

##### Antibacterial Activity of RWALC via the Disc Diffusion Method

The antibacterial activity of RWALC was investigated in the above-mentioned strains as described by Ahmed et al., with some modifications [80]. Bacterial suspensions were prepared in accordance with the 0.5 MacFarland turbidity standard (1.5 × 10^8^ CFU/mL) and spread onto MHA plates via sterile cotton swabs. Then, 5 mm × 5 mm cotton fabrics containing RW-AgNPs (RWALC) were placed on the agar. An untreated cotton fabric served as a negative control, whereas a positive control was established by adding 50 μL of 10 μg/mL ciprofloxacin for all strains or 5 μg/mL for *E. coli*. The plates were incubated at 37 °C for 24 h, and the zones of inhibition (ZOIs) were measured to assess antibacterial activity. The experiment was performed three times.

##### Effects of β-ME on the Antibacterial Activity of RWALC

The influence of β-ME (Sigma-Aldrich) on the antibacterial efficacy of RWALC was assessed via the previously described disc diffusion technique, with minor modifications [80]. The RWALC samples were preincubated with 10% β-ME solution for 5 min, rinsed with distilled water, and subsequently allowed to dry before application for the agar disc diffusion experiment, as described above.

## 4. Conclusions

This study demonstrated that RW-AgNPs possess significant antibacterial properties that retained when incorporated into textiles. These findings highlight the potential of using green nanotechnology in biomedicine to develop alternative or improved pharmaceutical products and antimicrobial therapies. The in situ synthesis of AgNPs offers notable advantages such as improved stability, increased uniformity, and cost efficiency. The RW-AgNPs showed impressive bactericidal activity against pathogens regarded as of high priority with low MIC and MBC, comparable to the conventional antibiotic, ciprofloxacin. RWALC could offer promise for treating bacterial infections, particularly in cases of chronic and infected wounds. However, additional research is needed to elucidate the mechanism of action underlying the antibacterial properties of RW-AgNPs and the RWALC and to assess their biocompatibility and cytotoxicity using relevant human cell models to ensure therapeutic efficacy at concentrations that are safe for healthy cells yet lethal to pathogenic bacteria. Furthermore, the antibacterial durability of RW-AgNPs impregnated cotton fabrics following repeated washing is crucial for practical textile applications. However, such evaluations were beyond the scope of the present study and warrant future investigations. Overall, RW-AgNPs and RWALC represent promising approaches for the development of innovative, accessible, and eco-friendly treatments for persistent bacterial infections.

## Figures and Tables

**Table 1:** The physicochemical properties of the optimised RW-AgNPs.

Analytical Technique	Physicochemical Properties	Measurement
UV-Vis analysis	SPR (nm)	408
	Absorbance at SPR	1.24
HR-TEM analysis	Core size distribution (nm)	5–11
	Average core size (nm)	9.2 ± 1.5
DLS analysis	Hydrodynamic size (d.nm)	104.3 ± 5.51
	PDI	0.344
	ζ-potential (mV)	−11 ± 1.5

**Table 2:** TPC of RW-E and RW-AgNPs.

Samples	TPC (μg GAE/mL)
RW-E	31.273 ± 2.89
RW-AgNPs	121.17 ± 7.93

**Table 3:** MIC and MBC values of RW-AgNPs against human pathogens.

Bacterial Strains	MIC (µg/mL)	MBC (µg/mL)
*S. aureus*	0.195	0.78
MRSA	1.56	3.125
*E. cloacae*	3.125	3.125
*E. coli*	0.78	0.78
*K. pneumoniae*	0.78	1.56
*P. aeruginosa*	3.125	6.25
*A. baumannii*	0.78	1.56

**Table 4:** ZOI of RWALC before and after incubation with β-ME against human pathogens.

Bacterial Strains	RWALC	RWALC with β-ME	Ciprofloxacin	Cotton Fabric
*S. aureus*	12.3 ± 1.2	0 ± 0	13.2 ± 3.0	0 ± 0
MRSA	13.8 ± 4.6	0 ± 0	11 ± 1.7	0 ± 0
*E. cloacae*	22.2 ± 1.9	0 ± 0	10 ± 3.0	0 ± 0
*E. coli*	19.2 ± 3.3	0 ± 0	17 ± 3.6	0 ± 0
*K. pneumoniae*	17 ± 2.7	0 ± 0	19.2 ± 1.4	0 ± 0
*P. aeruginosa*	14.7 ± 2.1	0 ± 0	12 ± 1.0	0 ± 0

## Data Availability

The original contributions presented in this study are included in the article/Supplementary Materials. Further inquiries can be directed to the corresponding authors.

## Supplementary Materials

The following supporting information can be downloaded at https://www.mdpi.com/article/10.3390/ijms27020952/s1.

## References

[1] Bertagnolio S., Dobreva Z., Centner C.M., Olaru I.D., Donà D., Burzo S., Huttner B.D., Chaillon A., Gebreselassie N., Wi T. (2024). WHO global research priorities for antimicrobial resistance in human health. Lancet Microbe.

[2] Ayres R.U. (2021). BiotechnologyBiotechnology and Human HealthHealth. The History and Future of Technology: Can Technology Save Humanity from Extinction?.

[3] Cano A., Ettcheto M., Espina M., Lopez-Machado A., Cajal Y., Rabanal F., Sanchez-Lopez E., Camins A., Garcia M.L., Souto E.B. (2020). State-of-the-art polymeric nanoparticles as promising therapeutic tools against human bacterial infections. J. Nanobiotechnol..

[4] Rappuoli R., Bloom D.E., Black S. (2017). Deploy vaccines to fight superbugs. Nature.

[5] Jorge P., Magalhães A.P., Grainha T., Alves D., Sousa A.M., Lopes S.P., Pereira M.O. (2019). Antimicrobial resistance three ways: Healthcare crisis, major concepts and the relevance of biofilms. FEMS Microbiol. Ecol..

[6] Tang K.W.K., Millar B.C., Moore J.E. (2023). Antimicrobial Resistance (AMR). Br. J. Biomed. Sci..

[7] Prestinaci F., Pezzotti P., Pantosti A. (2015). Antimicrobial resistance: A global multifaceted phenomenon. Pathog. Glob. Health.

[8] Lobanovska M., Pilla G. (2017). Focus: Drug development: Penicillin’s discovery and antibiotic resistance: Lessons for the future?. Yale J. Biol. Med..

[9] De Oliveira D.M., Forde B.M., Kidd T.J., Harris P.N., Schembri M.A., Beatson S.A., Paterson D.L., Walker M.J. (2020). Antimicrobial resistance in ESKAPE pathogens. Clin. Microbiol. Rev..

[10] Kaur A., Goyal D., Kumar R. (2018). Surfactant mediated interaction of vancomycin with silver nanoparticles. Appl. Surf. Sci..

[11] Zhang L., Bao M., Liu B., Zhao H., Zhang Y., Ji X., Zhao N., Zhang C., He X., Yi J. (2020). Effect of andrographolide and its analogs on bacterial infection: A review. Pharmacology.

[12] Llor C., Bjerrum L. (2014). Antimicrobial resistance: Risk associated with antibiotic overuse and initiatives to reduce the problem. Ther. Adv. Drug Saf..

[13] Hu D., Zou L., Gao Y., Jin Q., Ji J. (2020). Emerging nanobiomaterials against bacterial infections in postantibiotic era. VIEW.

[14] Efenberger-Szmechtyk M., Nowak A., Czyzowska A. (2021). Plant extracts rich in polyphenols: Antibacterial agents and natural preservatives for meat and meat products. Crit. Rev. Food Sci. Nutr..

[15] Brenes A., Viveros A., Chamorro S., Arija I. (2016). Use of polyphenol-rich grape by-products in monogastric nutrition. A review. Anim. Feed. Sci. Technol..

[16] Jara-Palacios M.J., Hernanz D., Cifuentes-Gomez T., Escudero-Gilete M.L., Heredia F.J., Spencer J.P. (2015). Assessment of white grape pomace from winemaking as source of bioactive compounds, and its antiproliferative activity. Food Chem..

[17] Gyawali R., Ibrahim S.A. (2014). Natural products as antimicrobial agents. Food Control.

[18] Alvarez-Martinez F.J., Barrajon-Catalan E., Micol V. (2020). Tackling Antibiotic Resistance with Compounds of Natural Origin: A Comprehensive Review. Biomedicines.

[19] Xu Y., Burton S., Kim C., Sismour E. (2016). Phenolic compounds, antioxidant, and antibacterial properties of pomace extracts from four Virginia-grown grape varieties. Food Sci. Nutr..

[20] Cheng V.J., Bekhit A.E.-D.A., McConnell M., Mros S., Zhao J. (2012). Effect of extraction solvent, waste fraction and grape variety on the antimicrobial and antioxidant activities of extracts from wine residue from cool climate. Food Chem..

[21] Serra A.T., Matias A.A., Nunes A.V.M., Leitão M.C., Brito D., Bronze R., Silva S., Pires A., Crespo M.T., San Romão M.V. (2008). In vitro evaluation of olive- and grape-based natural extracts as potential preservatives for food. Innov. Food Sci. Emerg. Technol..

[22] Katalinić V., Možina S.S., Skroza D., Generalić I., Abramovič H., Miloš M., Ljubenkov I., Piskernik S., Pezo I., Terpinc P. (2010). Polyphenolic profile, antioxidant properties and antimicrobial activity of grape skin extracts of 14 *Vitis vinifera* varieties grown in Dalmatia (Croatia). Food Chem..

[23] Langeveld W.T., Veldhuizen E.J., Burt S.A. (2014). Synergy between essential oil components and antibiotics: A review. Crit. Rev. Microbiol..

[24] Oliveira D.A., Salvador A.A., Smania A., Smania E.F., Maraschin M., Ferreira S.R. (2013). Antimicrobial activity and composition profile of grape (*Vitis vinifera*) pomace extracts obtained by supercritical fluids. J. Biotechnol..

[25] Friedman M. (2014). Antibacterial, antiviral, and antifungal properties of wines and winery byproducts in relation to their flavonoid content. J. Agric. Food Chem..

[26] Gianchecchi E., Fierabracci A. (2020). Insights on the Effects of Resveratrol and Some of Its Derivatives in Cancer and Autoimmunity: A Molecule with a Dual Activity. Antioxidants.

[27] Varoni E.M., Lo Faro A.F., Sharifi-Rad J., Iriti M. (2016). Anticancer molecular mechanisms of resveratrol. Front. Nutr..

[28] Ferraz da Costa D.C., Pereira Rangel L., Quarti J., Santos R.A., Silva J.L., Fialho E. (2020). Bioactive compounds and metabolites from grapes and red wine in breast cancer chemoprevention and therapy. Molecules.

[29] Saratale R.G., Saratale G.D., Ahn S., Shin H.S. (2021). Grape Pomace Extracted Tannin for Green Synthesis of Silver Nanoparticles: Assessment of Their Antidiabetic, Antioxidant Potential and Antimicrobial Activity. Polymers.

[30] Haleem A., Javaid M., Singh R.P., Rab S., Suman R. (2023). Applications of nanotechnology in medical field: A brief review. Glob. Health J..

[31] Zhang X.-F., Liu Z.-G., Shen W., Gurunathan S. (2016). Silver Nanoparticles: Synthesis, Characterization, Properties, Applications, and Therapeutic Approaches. Int. J. Mol. Sci..

[32] Almatroudi A. (2020). Silver nanoparticles: Synthesis, characterisation and biomedical applications. Open Life Sci..

[33] Soni R.A., Rizwan M.A., Singh S. (2022). Opportunities and potential of green chemistry in nanotechnology. Nanotechnol. Environ. Eng..

[34] Golpour M., Ebrahimnejad P., Gatabi Z.R., Najafi A., Davoodi A., Khajavi R., Alimohammadi M., Mousavi T. (2024). Green tea-mediated synthesis of silver nanoparticles: Enhanced anti-cancer activity and reduced cytotoxicity melanoma and normal murine cell lines. Inorg. Chem. Commun..

[35] Burange P.J., Tawar M.G., Bairagi R.A., Malviya V.R., Sahu V.K., Shewatkar S.N., Sawarkar R.A., Mamurkar R.R. (2021). Synthesis of silver nanoparticles by using *Aloe vera* and *Thuja orientalis* leaves extract and their biological activity: A comprehensive review. Bull. Natl. Res. Cent..

[36] Jiang Q., Yu S., Li X., Ma C., Li A. (2018). Evaluation of local anesthetic effects of Lidocaine-Ibuprofen ionic liquid stabilized silver nanoparticles in Male Swiss mice. J. Photochem. Photobiol. B.

[37] Ovais M., Khalil A.T., Raza A., Khan M.A., Ahmad I., Islam N.U., Saravanan M., Ubaid M.F., Ali M., Shinwari Z.K. (2016). Green synthesis of silver nanoparticles via plant extracts: Beginning a new era in cancer theranostics. Nanomedicine.

[38] Salve P., Vinchurkar A., Raut R., Chondekar R., Lakkakula J., Roy A., Hossain M.J., Alghamdi S., Almehmadi M., Abdulaziz O. (2022). An Evaluation of Antimicrobial, Anticancer, Anti-Inflammatory and Antioxidant Activities of Silver Nanoparticles Synthesized from Leaf Extract of *Madhuca longifolia* Utilizing Quantitative and Qualitative Methods. Molecules.

[39] Jiang T., Li Q., Qiu J., Chen J., Du S., Xu X., Wu Z., Yang X., Chen Z., Chen T. (2022). Nanobiotechnology: Applications in Chronic Wound Healing. Int. J. Nanomed..

[40] Gurunathan S., Han J.W., Kwon D.-N., Kim J.-H. (2014). Enhanced antibacterial and anti-biofilm activities of silver nanoparticles against Gram-negative and Gram-positive bacteria. Nanoscale Res. Lett..

[41] Kumar M., Curtis A., Hoskins C. (2018). Application of Nanoparticle Technologies in the Combat against Anti-Microbial Resistance. Pharmaceutics.

[42] Sibbald R.G., Contreras-Ruiz J., Coutts P., Fierheller M., Rothman A., Woo K. (2007). Bacteriology, inflammation, and healing: A study of nanocrystalline silver dressings in chronic venous leg ulcers. Adv. Ski. Wound Care.

[43] Novoa C.C., Tortella G., Seabra A.B., Diez M.C., Rubilar O. (2022). Cotton Textile with Antimicrobial Activity and Enhanced Durability Produced by L-Cysteine-Capped Silver Nanoparticles. Processes.

[44] Tang B., Kaur J., Sun L., Wang X. (2013). Multifunctionalization of cotton through in situ green synthesis of silver nanoparticles. Cellulose.

[45] Zhou Q., Lv J., Ren Y., Chen J., Gao D., Lu Z., Wang C. (2017). A green in situ synthesis of silver nanoparticles on cotton fabrics using *Aloe vera* leaf extraction for durable ultraviolet protection and antibacterial activity. Text. Res. J..

[46] Mpofu N.S., Mwasiagi J.I., Mecha C.A., Nganyi E.O. (2025). Evaluation of solanum tuberosum potato peel waste for use as an eco-friendly antibacterial finish for cotton fabrics. Res. J. Text. Appar..

[47] Hebeish A., El-Rafie M.H., El-Sheikh M.A., Seleem A.A., El-Naggar M.E. (2014). Antimicrobial wound dressing and anti-inflammatory efficacy of silver nanoparticles. Int. J. Biol. Macromol..

[48] Jain A., Kongkham B., Puttaswamy H., Butola B.S., Malik H.K., Malik A. (2022). Development of Wash-Durable Antimicrobial Cotton Fabrics by In Situ Green Synthesis of Silver Nanoparticles and Investigation of Their Antimicrobial Efficacy against Drug-Resistant Bacteria. Antibiotics.

[49] El-Naggar M.E., Shaheen T.I., Zaghloul S., El-Rafie M.H., Hebeish A. (2016). Antibacterial activities and UV protection of the in situ synthesized titanium oxide nanoparticles on cotton fabrics. Ind. Eng. Chem. Res..

[50] Hebeish A., El-Naggar M., Fouda M.M., Ramadan M., Al-Deyab S.S., El-Rafie M. (2011). Highly effective antibacterial textiles containing green synthesized silver nanoparticles. Carbohydr. Polym..

[51] Gonzalez-Ballesteros N., Rodriguez-Gonzalez J.B., Rodriguez-Arguelles M.C. (2018). Harnessing the wine dregs: An approach towards a more sustainable synthesis of gold and silver nanoparticles. J. Photochem. Photobiol. B.

[52] Delgado-Beleño Y., Martinez-Nuñez C.E., Cortez-Valadez M., Flores-López N.S., Flores-Acosta M. (2018). Optical properties of silver, silver sulfide and silver selenide nanoparticles and antibacterial applications. Mater. Res. Bull..

[53] Erasmus A. (2025). In Situ Green Synthesis of Red Wine Silver Nanoparticles for the Production of Antimicrobial Cotton Fabrics and the Investigation of Their Biomedical Effects. Master’s Thesis.

[54] Handayani W., Ningrum A.S., Imawan C. (2020). The Role of pH in Synthesis Silver Nanoparticles Using *Pometia pinnata* (Matoa) Leaves Extract as Bioreductor. J. Phys. Conf. Ser..

[55] Poudel B.K., Soe Z.C., Ruttala H.B., Gupta B., Ramasamy T., Thapa R.K., Gautam M., Ou W., Nguyen H.T., Jeong J.H. (2018). In situ fabrication of mesoporous silica-coated silver-gold hollow nanoshell for remotely controllable chemo-photothermal therapy via phase-change molecule as gatekeepers. Int. J. Pharm..

[56] Clogston J.D., Patri A.K., McNeil S.E. (2011). Zeta Potential Measurement. Characterization of Nanoparticles Intended for Drug Delivery.

[57] Makarov V.V., Love A.J., Sinitsyna O.V., Makarova S.S., Yaminsky I.V., Taliansky M.E., Kalinina N.O. (2014). “Green” nanotechnologies: Synthesis of metal nanoparticles using plants. Acta Naturae.

[58] Rajput S.K., Singh M.K., Shakyawar D.B. (2025). Herbal synthesis of silver nanoparticles for improved dyeing and UV protection of cotton fabric. Text. Res. J..

[59] Altemimi A., Lakhssassi N., Baharlouei A., Watson D.G., Lightfoot D.A. (2017). Phytochemicals: Extraction, Isolation, and Identification of Bioactive Compounds from Plant Extracts. Plants.

[60] Tooklang P., Audtarat S., Chaisen K., Thepsiri J., Chingsungnoen A., Jittabut P., Dasri T. (2024). Functionalization of silver nanoparticles coating cotton fabrics through hydrothermal synthesis for improved antimicrobial properties. Nano Express.

[61] Kaviya S., Santhanalakshmi J., Viswanathan B. (2012). Biosynthesis of silver nano-flakes by *Crossandra infundibuliformis* leaf extract. Mater. Lett..

[62] Thanh N.C., Pugazhendhi A., Chinnathambi A., Alharbi S.A., Subramani B., Brindhadevi K., Whangchai N., Pikulkaew S. (2022). Silver nanoparticles (AgNPs) fabricating potential of aqueous shoot extract of *Aristolochia bracteolata* and assessed their antioxidant efficiency. Environ. Res..

[63] Baliyan S., Mukherjee R., Priyadarshini A., Vibhuti A., Gupta A., Pandey R.P., Chang C.-M. (2022). Determination of Antioxidants by DPPH Radical Scavenging Activity and Quantitative Phytochemical Analysis of *Ficus religiosa*. Molecules.

[64] Mihailović V., Srećković N., Nedić Z.P., Dimitrijević S., Matić M., Obradović A., Selaković D., Rosić G., Katanić Stanković J.S. (2023). Green Synthesis of Silver Nanoparticles Using *Salvia verticillata* and *Filipendula ulmaria* Extracts: Optimization of Synthesis, Biological Activities, and Catalytic Properties. Molecules.

[65] Khuda F., Jamil M., Ali Khan Khalil A., Ullah R., Ullah N., Naureen F., Abbas M., Shafiq Khan M., Ali S., Muhammad Umer Farooqi H. (2022). Assessment of antioxidant and cytotoxic potential of silver nanoparticles synthesized from root extract of *Reynoutria japonica* Houtt. Arab. J. Chem..

[66] Perovic O., Singh-Moodley A., Govender N.P., Kularatne R., Whitelaw A., Chibabhai V., Naicker P., Mbelle N., Lekalakala R., Quan V. (2017). A small proportion of community-associated methicillin-resistant *Staphylococcus aureus* bacteraemia, compared to healthcare-associated cases, in two South African provinces. Eur. J. Clin. Microbiol. Infect. Dis..

[67] Vaiwala R., Sharma P., Ayappa G. (2022). Differentiating interactions of antimicrobials with Gram-negative and Gram-positive bacterial cell walls using molecular dynamics simulations. Biointerphases.

[68] Nandhini P., Kumar P., Mickymaray S., Alothaim A.S., Somasundaram J., Rajan M. (2022). Recent Developments in Methicillin-Resistant *Staphylococcus aureus* (MRSA) Treatment: A Review. Antibiotics.

[69] Oselusi S.O., Sibuyi N.R.S., Meyer M., Meyer S., Madiehe A.M. (2025). Phytofabrication of silver nanoparticles using *Ehretia rigida* leaf aqueous extract, their characterization, antioxidant and antimicrobial activities. Mater. Today Sustain..

[70] Balestri A., Cardellini J., Berti D. (2023). Gold and silver nanoparticles as tools to combat multidrug-resistant pathogens. Curr. Opin. Colloid. Interface Sci..

[71] Dube P., Meyer S., Madiehe A., Meyer M. (2020). Antibacterial activity of biogenic silver and gold nanoparticles synthesized from *Salvia africana-lutea* and *Sutherlandia frutescens*. Nanotechnology.

[72] Arshad H., Saleem M., Pasha U., Sadaf S. (2022). Synthesis of *Aloe vera*-conjugated silver nanoparticles for use against multidrug-resistant microorganisms. Electron. J. Biotechnol..

[73] Lethongkam S., Glaser J., Ammanath A.V., Voravuthikunchai S.P., Gotz F. (2023). In vitro and in vivo comparative analysis of antibacterial activity of green-synthesized silver nanoparticles. Biotechnol. J..

[74] Morones J.R., Elechiguerra J.L., Camacho A., Holt K., Kouri J.B., Ramírez J.T., Yacaman M.J. (2005). The bactericidal effect of silver nanoparticles. Nanotechnology.

[75] Lok C.-N., Ho C.-M., Chen R., He Q.-Y., Yu W.-Y., Sun H., Tam P.K.-H., Chiu J.-F., Che C.-M. (2007). Silver nanoparticles: Partial oxidation and antibacterial activities. JBIC J. Biol. Inorg. Chem..

[76] Pal S., Tak Y.K., Song J.M. (2007). Does the antibacterial activity of silver nanoparticles depend on the shape of the nanoparticle? A study of the Gram-negative bacterium *Escherichia coli*. Appl. Env. Microbiol..

[77] Mgijima T., Sibuyi N.R.S., Fadaka A.O., Meyer S., Madiehe A.M., Meyer M., Onani M.O. (2024). Wound healing effects of biogenic gold nanoparticles synthesized using red wine extracts. Artif. Cells Nanomed. Biotechnol..

[78] Aboyewa J.A., Sibuyi N.R.S., Goboza M., Murtz L.A., Oguntibeju O.O., Meyer M. (2022). Co-Treatment of Caco-2 Cells with Doxorubicin and Gold Nanoparticles Produced from *Cyclopia intermedia* Extracts or Mangiferin Enhances Drug Effects. Nanomaterials.

[79] Tyavambiza C., Elbagory A.M., Madiehe A.M., Meyer M., Meyer S. (2021). The antimicrobial and anti-inflammatory effects of silver nanoparticles synthesised from *Cotyledon orbiculata* aqueous extract. Nanomaterials.

[80] Ahmed O., Sibuyi N.R.S., Fadaka A.O., Madiehe A.M., Maboza E., Olivier A., Meyer M., Geerts G. (2022). Antimicrobial Effects of Gum Arabic-Silver Nanoparticles against Oral Pathogens. Bioinorg. Chem. Appl..

