# Supplementary material for: In Situ Green Synthesis of Red Wine Silver Nanoparticles on Cotton Fabrics and Investigation of Their Antibacterial Effects
Source: Int J Mol Sci. 2026 Jan 18;27(2):952. doi: 10.3390/ijms27020952 (PMC12842212; doi:10.3390/ijms27020952)
Supplement: Supplementary file 1 [file ijms-27-00952-s001.zip › ijms-4086750-supplementary.pdf]

Supplementary Information

In Situ Green Synthesis of Red Wine Silver Nanoparticles on Cotton Fabrics and Investigation of Their Antibacterial Effects

Alexandria Erasmus [1,2], Nicole Remaliah Samantha Sibuyi [2,3,*], Mervin Meyer [2] and Abram Madimabe Madiehe [1,2,*]

1 Nanobiotechnology Research Group, Department of Biotechnology, University of the Western Cape, Bellville 7535, South Africa
2 DSTI/Technology Innovation Agency Nanotechnology Platform, Department of Biotechnology, University of the Western Cape, Bellville 7535, South Africa
3 Health Platform, Advanced Materials Division, Mintek, Randburg 2194, South Africa
* Correspondence: nsibuyi@uwc.ac.za (N.R.S.S.); amadiehe@uwc.ac.za (A.M.M.); Tel.: +27-21-959-2735 (N.R.S.S.); +27-21-959-2468 (A.M.M.)

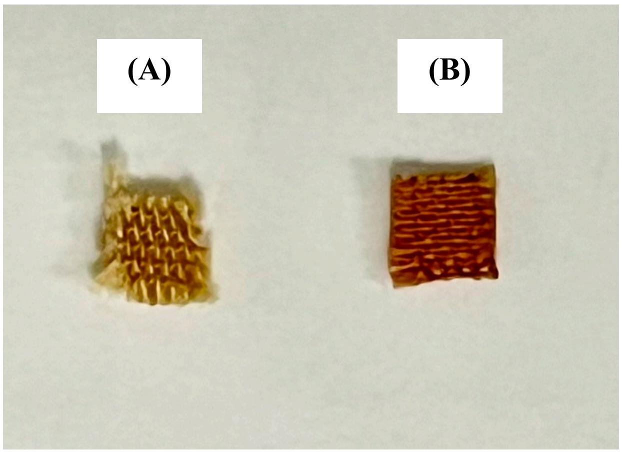

Figure S1: RWALC synthesised under hydrothermal conditions using 3 mM $AgNO_3$ and 6.25 mg/ml RW at (A) pH 10 and (B) at natural pH (4.3).

(A) **(B)** **(C)**

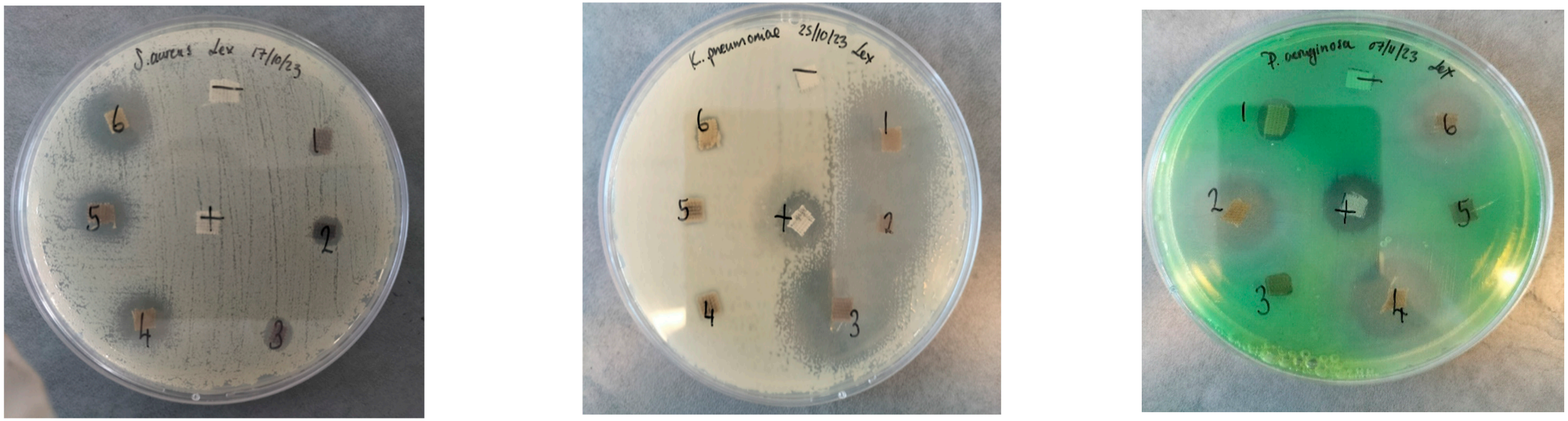

Figure S2: Antibacterial activity of RWALC using agar disc diffusion assay on selected bacteria. The bacteria were exposed to either RWALC (**4 – 6**) or RWALC preincubated with β-ME **(1-3)**. (A) *S. aureus*, (B) *K. pneumoniae*, and (C) *P. aeruginosa*.
